# The 150 most important questions in cancer research and clinical oncology series: questions 76–85: Edited by *Chinese Journal of Cancer*

**DOI:** 10.1186/s40880-017-0259-7

**Published:** 2017-11-20

**Authors:** Editorial Office of Chinese Journal of Cancer

**Affiliations:** Sun Yat-sen University Cancer Center, Guangzhou, 510060 Guangdong P. R. China

**Keywords:** Cancer cachexia, Animal models, Pancreatic cancer, Liver cancer, Antiangiogenic therapy, Tumor microenvironment, Tissue oxygenation, Anti-androgen drugs, Non-clinical information

## Abstract

Since the beginning of 2017, *Chinese Journal of Cancer* has published a series of important questions in cancer research and clinical oncology to promote cancer research and accelerate collaborations. In this article, 10 questions are presented as followed. Question 76. How to develop effective therapeutics for cancer cachexia? Question 77. How can we develop preclinical animal models to recapitulate clinical situations of cancer patients for more effective anti-cancer drug development? Question 78. How can we develop novel effective therapeutics for pancreatic cancer and hepatocellular carcinoma? Question 79. What are the true beneficial mechanisms of antiangiogenic therapy in cancer patients? Question 80. How to approach the complex mechanisms of interplay among various cellular and molecular components in the tumor microenvironment? Question 81. Can tissue oxygenation improve the efficacy of conventional chemotherapy on cancer? Question 82. Can tissue oxygenation improve the efficacy of radiotherapy on digestive system tumors including liver cancer? Question 83. Can we integrate metabolic priming into multimodal management of liver cancer? Question 84. Has the limit of anti-androgen strategy in prostate cancer treatment been reached by the new generation of anti-androgen drugs? Question 85. Can we identify individuals with early-stage cancers via analyzing their clinical and non-clinical information collected from social media, shopping history, and clinical, pathological, and molecular traces?

To promote cancer research and accelerate collaborations worldwide, *Chinese Journal of Cancer* has launched a program of publishing 150 most important questions in cancer research and clinical oncology [1]. Since the beginning of 2017, *Chinese Journal of Cancer* has published a series of important questions in cancer research and clinical oncology [2–11]. Questions 76–85 are selected and presented in this article. Our program of collecting and publishing the key questions is still ongoing. Please send your inspiring questions to Ms. Ji Ruan via email: ruanji@sysucc.org.cn.

## Question 76: How to develop effective therapeutics for cancer cachexia?

### Background and implications

Body weight loss, tissue wasting, appetite loss, asthenia, anemia, and metabolic changes are the main features of cancer cachexia. Cancer cachexia is a primary cause of death in cancer patients [12]. Dietary support alone cannot relieve cancer cachexia. Although cytokines, such as tumor necrosis factor-α (TNF-α) and interleukin-6 (IL-6) [13, 14], and metabolic changes, such as excessive fatty acid oxidation [15, 16], have been identified to contribute to cancer-associated systemic syndrome (CASS), their detailed molecular mechanisms remain largely unknown. To date, despite a few basic studies and a handful of clinical trials such as those applying anamorelin treatment for cachectic patients with non-small cell lung cancer (NSCLC) [17], practice guidelines for preventing and treating cancer cachexia are still lacking. To develop effective therapeutics for cancer cachexia, we need to elucidate the exact pathophysiological mechanisms of cancer cachexia and CASS. Clearly, this research direction will significantly improve life quality and prolong survival of cancer patients.

#### Submitter

Yihai Cao

#### Affiliation and email:

Department of Microbiology, Tumor and Cell Biology, Karolinska Institute, Stockholm 171 77, Sweden.

yihai.cao@ki.se

## Question 77: How can we develop preclinical animal models to recapitulate clinical situations of cancer patients for more effective anti-cancer drug development?

### Background and implications

Although commonly used in cancer research, the preclinical animal models show several distinct features as compared with cancer patients. (1) Genetic background: There are much more tumor heterogeneity and genetic background variations in cancer patients than in mouse models. (2) Outcome measurements: Survival is the gold standard for cancer patients, whereas in mouse models tumor size is often used as an indicator. (3) Tumor growth rate: In patients, cancers may slowly progress for years. However, in mouse models, tumors often grow much faster and may be more sensitive to anti-tumor therapy. (4) Therapy regimen: To test drug efficacy, therapies are often started on the early stage of tumor development in mouse models, whereas in human patients, drugs normally are given when advanced metastatic cancer occurs. New animal models that recapitulate clinical situations are needed for more effective anti-cancer drug development.

#### Submitter

Yihai Cao

#### Affiliation and email

Department of Microbiology, Tumor and Cell Biology, Karolinska Institute, Stockholm 171 77, Sweden.

yihai.cao@ki.se

## Question 78: How can we develop novel effective therapeutics for pancreatic cancer and hepatocellular carcinoma?

### Background and implications

To date, although several cancer types such as acute myeloid leukemia could be effectively treated, there are still great therapeutic challenges in lethal cancer types including pancreatic cancer and hepatocellular carcinoma (HCC). Pancreatic cancer, especially pancreatic adenocarcinoma (PDAC), is one of the most aggressive malignancies. HCC patients also have a poor prognosis, and the highest HCC incidence rates are reported in Asia [18]. For these lethal cancer types, conventional therapies showed limited survival benefit. Novel strategies, such as targeting cancer-associated fibroblasts (CAFs) in PDAC [19], have failed. Other than sorafenib, new targeted therapy for HCC has not been approved in the past decade [20]. Indeed, none of the established targeted therapy agents that have been used in other tumor types show similar effect on PDAC or HCC, indicating the intrinsic resistance of these two malignancies against already-approved anti-cancer drugs. Hence, targeting the unique mechanism of PDAC and/or HCC and preventing drug resistance should be the primary tasks in PDAC and HCC research.

#### Submitter

Yihai Cao

#### Affiliation and email

Department of Microbiology, Tumor and Cell Biology, Karolinska Institute, Stockholm 171 77, Sweden.

yihai.cao@ki.se

## Question 79: What are the true beneficial mechanisms of antiangiogenic therapy in cancer patients?

### Background and implications

In animal models, antiangiogenic therapy alone shows significant effect in controlling the growth of various cancer types. However, in cancer patients, antiangiogenic drugs must be combined with chemotherapy to be effective. The fundamental mechanisms that underlie the clinical benefit of antiangiogenic therapy in combination with traditional chemotherapy have not been fully elucidated. Several potential mechanisms have been proposed. (1) Normalization: Antiangiogenic drugs may induce tumor blood vessel remodeling, leading to a more normalized vasculature and improving drug delivery [21]. (2) Synergistic effect: Because chemotherapeutics primarily target tumor cells and antiangiogenic drugs target endothelial cells, the combination might lead to additive or synergistic antitumor activity [22]. (3) Off-tumor effects: Antiangiogenic drugs display off-tumor effects such as bone marrow vasculature alterations, and it might be beneficial for patients who suffer from cancer-associated systemic syndromes [23]. Discover the true mechanisms of antiangiogenic drugs will be valuable for new drug development and reduce drug resistance.

#### Submitter

Yihai Cao

#### Affiliation and email

Department of Microbiology, Tumor and Cell Biology, Karolinska Institute, Stockholm 171 77, Sweden.

yihai.cao@ki.se

## Question 80: How to approach the complex mechanisms of interplay among various cellular and molecular components in the tumor microenvironment?

### Background and implications

Tumor microenvironment consists of not only tumor cells but also various cellular components including endothelial cells, pericytes, fibroblasts, inflammatory cells, red blood cells, and necrotic cellular debris. Cancer cells often produce signaling molecules to manipulate host cells in the local microenvironment to facilitate their detachment, invasion, and metastasis. Although it is well known that several direct signaling pathways, such as the vascular endothelial growth factor (VEGF) signaling pathway, can promote angiogenesis for tumor growth and metastasis [24], the complicated interplays between tumor and host cells as well as among host cells might account for the majority of cancer invasion and metastasis. This complex network has never been fully understood. Although emerging evidence has unveiled several signaling pathways to be involved in cancer invasion and metastasis [25], a full landscape of tumor microenvironmental interactions is still longed for. The explorations in this direction would uncover more effective targets for controlling tumor progression and dissemination.

#### Submitter

Yihai Cao

#### Affiliation and email

Department of Microbiology, Tumor and Cell Biology, Karolinska Institute, Stockholm 171 77, Sweden.

yihai.cao@ki.se

## Question 81: Can tissue oxygenation improve the efficacy of conventional chemotherapy on cancer?

### Background and implications

Hypoxia is a common phenomenon in many types of solid tumors. Myo-inositol trispyrophosphate (ITPP) is a compound with anti-hypoxic properties by acting as an allosteric effector of hemoglobin to lower the affinity of hemoglobin for oxygen, subsequently enhancing the oxygen levels in the interstitial and tumor tissues [26, 27]. The promising anti-tumor effects of ITPP monotherapy have been shown in several animal models [26, 28]. The standard chemotherapy combination for colorectal cancer consists of folinic acid, fluorouracil, and oxaliplatin (FOLFOX). In a recent preclinical study, in comparison with the therapeutic efficacy of FOLFOX alone, the combination of ITPP with FOLFOX can extend the survival of the mice bearing liver metastases from colorectal cancer by 140% [29]. We therefore hypothesize that ITPP treatment could improve the efficacy of conventional chemotherapy. ITPP is currently used in phase IB/IIA clinical trials to evaluate the safety of the treatment and the efficacy of different doses. However, the biomarkers for the ITPP treatment response are to be revealed. The investigation in this field would provide us a new way to better control malignancies.

#### Submitters

Pierre-Alain Clavien^1^, Bostjan Humar^1^, Jean-Marie Lehn^2^

#### Affiliations and emails

^1^University Hospital Zurich, CH-8091 Zurich, Switzerland; ^2^Université de Strasbourg, F-67081 Strasbourg, France

clavien@access.uzh.ch, bostjan.humar@usz.ch, lehn@unistra.fr

## Question 82: Can tissue oxygenation improve the efficacy of radiotherapy on digestive system tumors including liver cancer?

### Background and implications

The therapeutic efficacy of ionizing radiation depends on high oxygen levels to induce double-strand DNA breaks in proliferating tumor cells. However, hypoxia in the central area of tumor nests could diminish the therapeutic efficacy of radiation. In our unpublished study, ITPP could enhance the growth inhibitory effects of radiation on squamous cell carcinoma subcutaneously implanted in nude mice. Based on our previous findings that ITPP monotherapy could effectively suppress and eradicate hepatoma in rats via increasing the tumor oxygen level [26], we herein hypothesize that ITPP can improve the efficacy of radiotherapy on digestive system tumors including liver cancer. We are currently testing an ITPP-radiotherapy combination in a syngeneic mouse model of liver metastases from colorectal cancer. The knowledge accumulated in this field could further enhance the efficacy of radiotherapy on gastrointestinal tumors.

#### Submitters

Pierre-Alain Clavien^1^, Bostjan Humar^1^, Jean-Marie Lehn^2^

#### Affiliations and emails

^1^University Hospital Zurich, CH-8091 Zurich, Switzerland; ^2^Université de Strasbourg, F-67081 Strasbourg, France

clavien@access.uzh.ch, bostjan.humar@usz.ch, lehn@unistra.fr

## Question 83: Can we integrate metabolic priming into multimodal management of liver cancer?

### Background and implications

It has been found that high dietary glycemic load associates with an elevated risk of hepatocellular carcinoma (HCC) [30], suggesting the involvement of high glucose levels in cancer initiation. High fasting glucose levels also associate with increased mortalities of several cancer types, with the strongest association in pancreatic cancer, followed by the cancers of the esophagus, liver, and colon [31], suggesting the involvement of high glucose levels in cancer progression. A recent, interesting study has reported that in a DEN-induced mouse HCC model, sugar diet increases, while fat diet reduces tumor burden [32]. Cancer has been the top one killer in many countries. In the most comprehensive epidemiological study thus far [33], the risk of all-cause mortality excluding cardiovascular disease is increased with more intake of carbohydrates and is reduced with more intake of fat (including saturated and unsaturated fat).

We previously have reported that fat provides energy for liver regeneration [34], and omega-3 fatty acids can mitigate steatosis (a HCC risk factor), protect the liver tissue from ischemia, promote liver regeneration, and reduce liver cancer burden [35]. Subsequently, we found that exercise or adenosine 5′-monophosphate (AMP)-activated protein kinase (AMPK) activators can promote fat oxidation, mitigate steatosis, protect the liver tissue from ischemia, and promote liver regeneration. As an indirect AMPK activator, metformin has been found to be able to reduce the mortality from HCC and colorectal cancer [36, 37].

We herein hypothesize that by promoting fat oxidation (e.g., dietary fat plus carnitine plus AMPK activator) while reducing carbohydrate intake, we can improve the outcome of liver cancer patients who undergo surgery. It therefore appears worthy of envisaging clinical trials combining metabolic priming with anti-cancer treatments.

#### Submitters

Pierre-Alain Clavien^1^, Bostjan Humar^1^, Jean-Marie Lehn^2^

#### Affiliations and emails

^1^University Hospital Zurich, CH-8091 Zurich, Switzerland; ^2^Université de Strasbourg, F-67081 Strasbourg, France

clavien@access.uzh.ch, bostjan.humar@usz.ch, lehn@unistra.fr

## Question 84: Has the limit of anti-androgen strategy in prostate cancer treatment been reached by the new generation of anti-androgen drugs?

### Background and implication

Although immune therapy recently made significant progress in the treatment of several types of cancer, anti-androgen signaling drugs remain as the first choice and most effective treatment for early-stage prostate cancer. However, the anti-androgen treatment will eventually fail as tumor cells adapt to androgen-independent growth in the later stage of prostate cancer. The new generation of anti-androgen drugs have been developed, such as MDV3100 (Enzalutamide), showing a therapeutic effect of extending the overall survival of the patients with androgen-refractory, late-stage prostate cancer up to 6 months. The question is whether the limit of anti-androgen strategy has been reached. Cell-based assay shows that even the latest anti-androgen drug (Enzalutamide) can only repress 30% of full agonist activity, suggesting that there are still spaces for the new generation of anti-androgen drugs to improve. Therefore, the exploration in this direction is promising.

#### Submitter

Yuanzheng He

#### Affiliation and email

Van Andel Research Institute, Grand Rapids, MI 49503, USA

ajian.he@vai.org

## Question 85: Can we identify individuals with early-stage cancers via analyzing their clinical and non-clinical information collected from social media, shopping history, and clinical, pathological, and molecular traces?

### Background and implication

There has recently been an unprecedented growth in the amount of data being generated by (and for) the ordinary public in online and digital social spheres as well as in the clinical domains. Simultaneously, there have been significant technological advances off late in terms of machine-learning technologies, graphics processing unit, cloud computing, and data storage. These concurrent developments in biomedical, social media, and computing technologies now make it possible to explore trends and patterns of early-stage cancers in a systematic data-driven manner. In today’s world, we are leaving digital traces behind us in both clinical and non-clinical contexts on a regular basis. While there is general consensus that an intelligent combination of multiple, independent sources of data from social media, online search and shopping histories, and clinical, pathological, and molecular data can provide a more powerful way of detecting early-stage cancers as compared with any of these sources alone, it is less clear how to computationally combine these disparate pieces of unstructured information to create an integrated detector with high sensitivity and specificity. This enormous challenge also presents a massive opportunity to develop novel artificial intelligence (AI)-based algorithms to integrate and analyze the data from different sources for indentifying individuals with early-stage cancers.

#### Submitter

Nasir Rajpoot

#### Affiliation and email

Department of Computer Science, University of Warwick, Coventry, CV4 7AL, England, UK

N.M.Rajpoot@warwick.ac.uk

## References

[1] Qian CN, Zhang W, Xu RH (2016). Defeating cancer: the 150 most important questions in cancer research and clinical oncology. Chin J Cancer.

[2] Wee JT, Poh SS (2017). The most important questions in cancer research and clinical oncology. Question 1. Could the vertical transmission of human papilloma virus (HPV) infection account for the cause, characteristics, and epidemiology of HPV-positive oropharyngeal carcinoma, non-smoking East Asian female lung adenocarcinoma, and/or East Asian triple-negative breast carcinoma?. Chin J Cancer.

[3] Venniyoor A (2017). The most important questions in cancer research and clinical oncology—Question 2–5. Obesity-related cancers: more questions than answers. Chin J Cancer.

[4] Chinese Journal of Cancer (2017). The 150 most important questions in cancer research and clinical oncology series: questions 6–14. Chin J Cancer.

[5] Chinese Journal of Cancer (2017). The 150 most important questions in cancer research and clinical oncology series: questions 15–24. Chin J Cancer..

[6] Chinese Journal of Cancer (2017). The 150 most important questions in cancer research and clinical oncology series: questions 25–30. Chin J Cancer..

[7] Chinese Journal of Cancer (2017). The 150 most important questions in cancer research and clinical oncology series: questions 31–39. Chin J Cancer..

[8] Chinese Journal of Cancer (2017). The 150 most important questions in cancer research and clinical oncology series: questions 40–49. Chin J Cancer..

[9] Chinese Journal of Cancer (2017). The 150 most important questions in cancer research and clinical oncology series: questions 50–56. Chin J Cancer..

[10] Chinese Journal of Cancer (2017). The 150 most important questions in cancer research and clinical oncology series: questions 57–66. Chin J Cancer..

[11] Chinese Journal of Cancer (2017). The 150 most important questions in cancer research and clinical oncology series: questions 67–75. Chin J Cancer..

[12] Tisdale MJ (2002). Cachexia in cancer patients. Nat Rev Cancer.

[13] Kayacan O (2006). Impact of TNF-alpha and IL-6 levels on development of cachexia in newly diagnosed NSCLC patients. Am J Clin Oncol.

[14] Oliff A (1988). The role of tumor necrosis factor (cachectin) in cachexia. Cell.

[15] Fukawa T (2016). Excessive fatty acid oxidation induces muscle atrophy in cancer cachexia. Nat Med.

[16] Qian CN (2016). The rationale for preventing cancer cachexia: targeting excessive fatty acid oxidation. Chin J Cancer.

[17] Currow DC, Skipworth RJ (2017). The emerging role of anamorelin hydrochloride in the management of patients with cancer anorexia-cachexia. Future Oncol.

[18] McGlynn KA, London WT (2011). The global epidemiology of hepatocellular carcinoma: present and future. Clin Liver Dis.

[19] Ozdemir BC (2015). Depletion of carcinoma-associated fibroblasts and fibrosis induces immunosuppression and accelerates pancreas cancer with reduced survival. Cancer Cell.

[20] Llovet JM, Hernandez-Gea V (2014). Hepatocellular carcinoma: reasons for phase III failure and novel perspectives on trial design. Clin Cancer Res.

[21] Jain RK (2005). Normalization of tumor vasculature: an emerging concept in antiangiogenic therapy. Science.

[22] Plum SM (2003). Synergistic activity of recombinant human endostatin in combination with adriamycin: analysis of in vitro activity on endothelial cells and in vivo tumor progression in an orthotopic murine mammary carcinoma model. Clin Cancer Res.

[23] Cao Y (2010). Off-tumor target–beneficial site for antiangiogenic cancer therapy?. Nat Rev Clin Oncol.

[24] Ferrara N (2002). VEGF and the quest for tumour angiogenesis factors. Nat Rev Cancer.

[25] Yang Y (2016). The PDGF-BB-SOX7 axis-modulated IL-33 in pericytes and stromal cells promotes metastasis through tumour-associated macrophages. Nat Commun.

[26] Aprahamian M, Bour G, Akladios CY, Fylaktakidou K, Greferath R, Soler L (2011). Myo-InositolTrisPyroPhosphate treatment leads to HIF-1alpha suppression and eradication of early hepatoma tumors in rats. Chem bio chem Eur J Chem Biol.

[27] Fylaktakidou KC, Lehn JM, Greferath R, Nicolau C (2005). Inositol tripyrophosphate: a new membrane permeant allosteric effector of haemoglobin. Bioorg Med Chem Lett.

[28] Derbal-Wolfrom L, Pencreach E, Saandi T, Aprahamian M, Martin E, Greferath R (2013). Increasing the oxygen load by treatment with myo-inositol trispyrophosphate reduces growth of colon cancer and modulates the intestine homeobox gene Cdx2. Oncogene.

[29] Limani P, Linecker M, Kachaylo E, Tschuor C, Kron P, Schlegel A (2016). Antihypoxic potentiation of standard therapy for experimental colorectal liver metastasis through myo-inositol trispyrophosphate. Clin Cancer Res Off J Am Assoc Cancer Res.

[30] Rossi M, Lipworth L, Maso LD, Talamini R, Montella M, Polesel J (2009). Dietary glycemic load and hepatocellular carcinoma with or without chronic hepatitis infection. Ann Oncol Off J Eur Soc Med Oncol.

[31] Jee SH, Ohrr H, Sull JW, Yun JE, Ji M, Samet JM (2005). Fasting serum glucose level and cancer risk in Korean men and women. JAMA.

[32] Healy ME, Chow JD, Byrne FL, Breen DS, Leitinger N, Li C (2015). Dietary effects on liver tumor burden in mice treated with the hepatocellular carcinogen diethylnitrosamine. J Hepatol.

[33] Dehghan M, Mente A, Zhang X, Swaminathan S, Li W, Mohan V (2017). Associations of fats and carbohydrate intake with cardiovascular disease and mortality in 18 countries from five continents (PURE): a prospective cohort study. Lancet.

[34] Kachaylo E, Tschuor C, Calo N, Borgeaud N, Ungethum U, Limani P (2017). PTEN down-regulation promotes beta-oxidation to fuel hypertrophic liver growth after hepatectomy in mice. Hepatology.

[35] Linecker M, Limani P, Kambakamba P, Kron P, Tschuor C, Calo N (2017). Omega-3 fatty acids protect fatty and lean mouse livers after major hepatectomy. Ann Surg.

[36] He XK, Su TT, Si JM, Sun LM (2016). Metformin is associated with slightly reduced risk of colorectal cancer and moderate survival benefits in diabetes mellitus: a meta-analysis. Medicine..

[37] Ma SJ, Zheng YX, Zhou PC, Xiao YN, Tan HZ (2016). Metformin use improves survival of diabetic liver cancer patients: systematic review and meta-analysis. Oncotarget.

